# The Effect of Dexmedetomidine on Lumbar Epidural Injection for Failed Back Surgery Syndrome

**DOI:** 10.1155/2016/7198048

**Published:** 2016-08-17

**Authors:** Ashraf Eskandr, Sadik Abdel Maseeh

**Affiliations:** Department of Anaesthesia, ICU and Pain Therapy, Faculty of Medicine, Menoufiya University, Shibin El-Koom 32518, Egypt

## Abstract

*Purpose*. Failed back surgery syndrome is a chronic pain condition requiring rapid, effective, and efficient management. This study evaluates the effect of adding dexmedetomidine to lumbar epidural steroids in patients with failed back surgery syndrome.* Methods*. Fifty patients suffering from failed back surgery syndrome were randomly assigned to one of two groups, receiving an epidural injection of 20 mL of either a mixture of betamethasone (14 mg) and bupivacaine 0.5 mg (group C) or a mixture of betamethasone (14 mg), bupivacaine 0.5 mg, and dexmedetomidine (0.5 *μ*g/kg) (group D) adjusted to the volume with normal saline. The effect was evaluated using visual analogue scale (VAS), analgesic requirement, and Oswestry disability index 2 weeks, 4 weeks, 8 weeks, and 12 weeks after injection.* Results*. VAS and ibuprofen consumption showed a significant reduction in group D. The Oswestry disability index was significantly improved in group D. There were no records of hypotension, bradycardia, sedation, or hypoxemia in both groups.* Conclusion*. The present study demonstrated potential safe and effective usage of adding dexmedetomidine to epidural steroid to control pain in patients with failed back surgery syndrome.

## 1. Introduction

Failed back surgery syndromes (FBSS) occur in 5–40% of patients that underwent a back surgery [1] due to spinal stenosis, spinal segment degeneration, disc ruptures, recurred disc herniation, disc fragments remaining after surgery, epidural scars, facet joint pain, sacroiliac joint pain, spinal segment instability, and so forth [2]. Failed back surgery syndrome is a chronic pain condition which has a considerable effect on the patient and health care system [3]. So, finding an effective, rapid, and efficient method for pain control and treatment is highly desirable [4]. Lumbar epidural injection of long-acting steroids is commonly used to alleviate low back pain. The duration of analgesia varies among patients with FBSS and ranges from 15 days up to 180 days [4]. The mechanism of action of steroids in pain reduction following epidural injection is not well understood. However, the anti-inflammatory effects are the most probable mechanism, wherein the levels of tissue phospholipase and prostaglandins are decreased by steroids [5]. Alpha-2 agonists had been shown to reduce chronic allodynia in rat animal models [6], most probably through blocking pre- and postsynaptic *α*-2 receptors [7]. The addition of clonidine (*α*-2 agonist) to epidural steroid has been used to treat chronic intractable postthoracotomy pain [8].

We designed the present double-blind and randomized study using dexmedetomidine as an adjunct to epidural steroids to examine the effect of dexmedetomidine on pain, amount of analgesics, and activity of patients suffering FBSS.

## 2. Materials and Methods

After obtaining approval from the institutional ethics committee and written informed consent, 50 patients, 40–70 years old of both sexes, ASA physical status I–III, suffering from FBSS were included in this prospective, double-blind, randomized controlled study at the Menoufiya University outpatient pain clinic. The study protocol was registered with the Pan-African Clinical Trial Registry (http://www.pactr.org/). Patients were excluded if they suffered from a sacroiliac joint disease, facet joint arthritis revealed by magnetic resonant imaging, severe cardiopulmonary disease, uncontrolled diabetes, morbid obesity (body mass index ≥ 40 Kg/m^2^), addiction, infection, and coagulation disorders and if patients were on opioid medications for pain management before or during the study period. After explaining study protocol and visual analogue score (VAS) graduated from zero (no pain) to 10 cm (worst pain) to the patients, they were randomly assigned by using closed envelope technique to one of two groups. Group C (control group) received an epidural injection of 20 mL of a mixture of betamethasone (14 mg) and bupivacaine (0.5 mg) adjusted to the volume with normal saline. Group D (dexmedetomidine group) received an epidural injection of 20 mL of a mixture of betamethasone (14 mg), bupivacaine (0.5 mg), and dexmedetomidine (0.5 *μ*g/kg) adjusted to the volume with normal saline. The epidural injection was administered with the patient in a lateral recumbent position, while his/her neck, pelvis, and knees were bent. The skin and subcutaneous tissues were anesthetized by injecting 1-2 mL of lidocaine 2% between L2 and L5 spinous processes (identified by fluoroscopy) and a 20-gauge Touhy needle was inserted approximately 2-3 cm so that the needle went into the interspinal ligament. Then, a syringe containing air was attached to the needle and the needle was inserted slowly, 1-2 mm at a time until no resistance was felt. Afterward, 2 mL of nonionic radiocontrast was injected into the location of the needle to identify that the contrast was diffusing in the shape of thin strings toward the top of the image. When the needle was identified in the epidural space through the interspinal ligament approach method, the study drugs were injected. The patients were then transferred to the Peri-Anesthesia Care Unit (PACU) for monitoring vital signs, pain levels, and possible neurological and other adverse events for 60–90 minutes. They were then discharged home (4 hours after the procedure) in the care of a responsible adult and advised not to drive for 24 hours. The pain intensity was evaluated by visual analogue scale scores. As per the patient's request to analgesia when VAS was greater than or equal to 4, oral 200 mg ibuprofen tablet was given and the total amount of nonsteroidal anti-inflammatory drug (NSAID) per week was calculated and recorded. The effects of the procedures were evaluated by measuring the visual analogue scale (VAS) and Oswestry disability index (ODI) before the procedures then 2 weeks, 4 weeks, 8 weeks, and 12 weeks after the procedure. The incidence of complications was recorded, including the occurrence of hypotension (systolic blood pressure < 90 mmHg), bradycardia (pulse rate < 50/minute), extensive sedation (any sedation score above 1; 1 = sedated, but easily to be aroused), or hypoxemia (oxygen saturation below 90%). In a case of marked hypotension, rapid infusion of 500 mL crystalloids and ephedrine 5 mg was given, and heart rate less than 50 beats/min was treated with 0.3 mg atropine.

### 2.1. Statistical Analysis

The sample size was calculated based on results of previous studies, using a power of 85% and an *α* value 0.05. We assumed that the minimum difference of back pain score was 20% and 95% confidence interval. The sample size was calculated to be 23 patients so we decided to include 25 patients in each group in this study. We used GraphPad Stat-Mate version 2 statistics program for power analysis.

The collected data were analyzed by Statistical Package for Social Science (SPSS) version 16. Parametric data were expressed as mean ± SD. The comparison of the mean ± SD of two groups was done using the paired and unpaired Student's *t*-test (analgesic requirement, Oswestry disability index, age, weight, and height). Nonparametric data was expressed as a number of patients or median (interquartile range). Determining the extent of a single observed series of proportions and difference from a theoretical or expected distribution was done using the Chi-square test (sex) and Mann-Whitney *U* test (VAS). *P* value < 0.05 was considered statistically significant.

## 3. Results

The study was performed on 50 patients randomly divided into 2 equal groups. Patients characteristics were similar in the 2 groups regarding the demographic data (age, sex, height, and weight) (Table 1). For VAS of pain, there was a significant decrease in dexmedetomidine group compared to control group at 2, 4, 8, and 12 weeks after the procedure (Table 2). The mean consumption of NSAID during the study period was also significantly lower in dexmedetomidine group more than in control group (Table 3). Furthermore, there was a significant improvement in the quality of life and the levels of personal activity in the dexmedetomidine group as shown by the significant reduction of ODI in the group during the study period (Table 4). As regards side effects of dexmedetomidine injection, there was no recorded incident of hypotension, bradycardia, sedation, and hypoxemia.

## 4. Discussion

FBSS is a major concern in pain therapy. Although the exact incidence remains unknown, it is estimated to be as high as 60% of patients who have had back surgeries [9]. In this study, we used dexmedetomidine as a novel adjuvant to epidural steroids to control pain in patients with failed back surgery. The study showed that addition of dexmedetomidine to epidural steroids results in superior pain control. This is evidenced by the decrease in VAS of back pain, along with a decrease in the total required dose of NSAID and a better lifestyle as documented by reduced ODI in patients suffering FBSS.

Due to the fact that dexmedetomidine has a high selectivity to *α*2 adrenergic receptor binding, it is widely used as a systemic analgesic adjuvant [10]. Furthermore, dexmedetomidine has been investigated as an adjuvant to local anesthetics in regional anesthesia and analgesia [11, 12]. Since the analgesic effect of *α*2-AR agonists is mostly mediated at the spinal level, neuraxial administration was chosen for the dexmedetomidine adjuvant. Moreover, its high lipophilicity allows for rapid absorption into the cerebrospinal fluid and binding to the spinal cord *α*2-AR [13].

Many studies have used dexmedetomidine safely in a dose of 1-2 *μ*g/kg administered either epidurally [14–16] or caudally [12, 17–19] for the management of postoperative pain. In the present study, we used dexmedetomidine in a dose of 0.5 *μ*g/kg added to epidural betamethasone due to short term follow-up of the patients as they were discharged home 4 hours after the procedure.

In the current study, we evaluated the effectiveness of the analgesic effect of long-acting steroid preparation, betamethasone, mixed with dexmedetomidine. The primary efficacy outcome parameters were a reduction in the intensity of pain after treatment with the epidural injection. Also, NSAID doses were significantly reduced when dexmedetomidine was added to the epidural steroid. Life style was improved in dexmedetomidine group as reported by the significant decrease in SDI. These results are consistent with the results of other studies using dexmedetomidine as an adjunct in the treatment of chronic pain syndromes in human [20–22], such as Lee and colleagues [20] who reported the antihyperalgesic effect of dexmedetomidine on patients undergoing a laparoscopic assisted vaginal hysterectomy. Additionally, Jain and others [21] found that the perioperative infusion of dexmedetomidine has a pivotal role in attenuating the incidence and severity of chronic pain and improving the quality of life in cases undergoing breast cancer surgery. Nama Sharanya et al. [22] reported the use of dexmedetomidine as an adjuvant with subanesthetic intravenous ketamine was effective in managing complex regional pain syndrome. This also agrees with the experimental work of Li and coworkers [23] who studied the analgesic effect of dexmedetomidine on naturopathic pain, as well as with Farghaly and colleagues [24] who studied the effects of intraperitoneal dexmedetomidine alone and in combination with tramadol or amitriptyline in a neuropathic pain model.

The same results of the present study were found by other studies performed by using another *α*2-AR agonist clonidine administered epidurally to control chronic pain. Lavand'homme and De Kock found that epidural or intrathecal clonidine added to local anesthetic may also prevent the development of chronic postsurgical pain and hyperalgesia after major abdominal surgery [25]. Lauretti et al. reported the similar efficacy of epidural clonidine and ketamine in chronic cancer pain [26]. Moreover, Ayad and El Masry [8] reported in a pilot study that a combination of epidural steroid and clonidine may provide pain relief in chronic postthoracotomy pain.

No significant adverse effects in the form of hypotension, bradycardia, sedation, or hypoxemia occurred and did not affect the time of hospital discharge of patients. All patients were discharged after 4 hours of the injection.

In conclusion, the present study demonstrated a potential safe and effective usage of adding dexmedetomidine to epidural steroid to control pain in patient with failed back surgery syndrome, as reflected by the significant reduction of pain intensity, reduction of NSID doses, and better quality of life with no significant adverse effects.

## Figures and Tables

**Table 1:** Demographic data.

Character	Group C (*n* = 25)	Group D (*n* = 25)	*P* value
Age (years)	52 ± 7.36	52.52 ± 8.36	0.816
Sex (F/M)	14/11	16/9	0.773
Weight (kg)	86.56 ± 7.84	87.32 ± 7.69	0.731
Height (cm)	163.96 ± 5.99	163.2 ± 6.12	0.659

Group C: control group; group D: dexmedetomidine group; *n* = number of patients; M: male; F: female. Data were expressed as mean ± standard deviation and number of patients.

**Table 2:** Visual analogue scale of back pain.

Time	Group C (*n* = 25)	Group D (*n* = 25)	*P* value
Before	5 (4–8)	5 (4–8)	0.973
Week 2	4 (3–7)	3 (1–5)^*∗*^	0.028
4	4 (3–7)	3 (2–6)^*∗*^	0.049
8	4 (3–7)	4 (2–6)^*∗*^	0.045
12	4 (3–7)	4 (2–6)^*∗*^	0.023

Group C: control group; group D: dexmedetomidine group; *n* = number of patients. Data were expressed as median (interquartile range). ^*∗*^ *P* < 0.05: significant.

**Table 3:** Ibuprofen (NSAID) consumption (mg/week).

Time	Group C (*n* = 25)	Group D (*n* = 25)	*P* value
Before	1412 ± 358.77	1408 ± 358.14	0.969
2	1220 ± 320.16	1032 ± 260.83^*∗*^	0.027
4	1268 ± 321.85	1064 ± 288.71^*∗*^	0.013
8	1286 ± 322.02	1056 ± 262.01^*∗*^	0.008
12	1290 ± 322.38	1020 ± 260.66^*∗*^	0.002

Group C: control group; group D: dexmedetomidine group; *n* = number of patients. Data were expressed as mean ± standard deviation. ^*∗*^ *P* < 0.05: significant.

**Table 4:** Oswestry disability index (ODI).

Time	Group C (*n* = 25)	Group D (*n* = 25)	*P* value
Before	24.16 ± 8.74	23.12 ± 8.66	0.674
2	22.01 ± 7.93	16.11 ± 7.93^*∗*^	0.011
4	19.93 ± 7.19	15.54 ± 8.01^*∗*^	0.047
8	19.87 ± 7.12	15.1 ± 7.42^*∗*^	0.025
12	19.52 ± 6.19	15.33 ± 7.41^*∗*^	0.035

Group C: control group; group D: dexmedetomidine group; *n* = number of patients. Data were expressed as mean ± standard deviation. ^*∗*^ *P* < 0.05: significant.

## References

[1] Kim S. B., Lee K. W., Lee J. H., Kim M. A., An B. W. (2012). The effect of hyaluronidase in interlaminar lumbar epidural injection for failed back surgery syndrome. *Annals of Rehabilitation Medicine*.

[2] Yang H. S., Lee S. H., Ryu C. H., Lee J. Y., Bae J. H. (2004). Correlation of the clinical factors and gait parameters in failed back surgery syndrome. *Journal of Korean Academy of Rehabilitation Medicine*.

[3] Chan C.-W., Peng P. (2011). Failed back surgery syndrome. *Pain Medicine*.

[4] Rahimzadeh P., Sharma V., Imani F. (2014). Adjuvant hyaluronidase to epidural steroid improves the quality of analgesia in failed back surgery syndrome: a prospective randomized clinical trial. *Pain Physician*.

[5] Geurts J. W., Kallewaard J.-W., Richardson J., Groen G. J. (2002). Targeted methylprednisolone acetate/hyaluronidase/clonidine injection after diagnostic epiduroscopy for chronic sciatica: a prospective, 1-year follow-up study. *Regional Anesthesia and Pain Medicine*.

[6] Buvanendran A., Kroin J. S., Kerns J. M., Nagalla S. N. K., Tuman K. J. (2004). Characterization of a new animal model for evaluation of persistent postthoracotomy pain. *Anesthesia and Analgesia*.

[7] Julião M.-D. C., Lauretti G. R. (2000). Low-dose intrathecal clonidine combined with sufentanil as analgesic drugs in abdominal gynecological surgery. *Journal of Clinical Anesthesia*.

[8] Ayad A. E., El Masry A. (2012). Epidural steroid and clonidine for chronic intractable post-thoracotomy pain: A Pilot Study. *Pain Practice*.

[9] Manchikanti L., Singh V., Cash K. A., Pampati V., Datta S. (2008). Preliminary results of a randomized, equivalence trial of fluoroscopic caudal epidural injections in managing chronic low back pain: part 3—Post surgery syndrome. *Pain Physician*.

[10] Kosharskyy B., Almonte W., Shaparin N., Pappagallo M., Smith H. (2013). Intravenous infusions in chronic pain management. *Pain Physician*.

[11] Sukhminder J. S., Jasbir K., Vikramjit A., Sachin G., Amarjit S., Parmar S. S. (2011). Comparative evaluation of dexmedetomidine and fentanyl for epidural analgesia in lower limb orthopedic surgeries. *Indian Journal of Anaesthesia*.

[12] Saadawy I., Boker A., Elshahawy M. A. (2009). Effect of dexmedetomidine on the characteristics of bupivacaine in a caudal block in pediatrics. *Acta Anaesthesiologica Scandinavica*.

[13] Grosu I., Lavand'homme P. (2010). Use of dexmedetomidine for pain control. *F1000 Medicine Reports*.

[14] Bajwa S. J. S., Bajwa S. K., Kaur J. (2011). Dexmedetomidine and clonidine in epidural anaesthesia: a comparative evaluation. *Indian Journal of Anaesthesia*.

[15] Bajwa S. J. S., Arora V., Kaur J., Singh A., Parmar S. S. (2011). Comparative evaluation of dexmedetomidine and fentanyl for epidural analgesia in lower limb orthopedic surgeries. *Saudi Journal of Anaesthesia*.

[16] Selim M. F., Elnabtity A. M., Hasan A. M. (2012). Comparative evaluation of epidural bupivacaine—dexmedetomidine and bupivacaine-fentanyl on Doppler velocimetry of uterine and umbilical arteries during labour. *Journal of Prenatal Medicine*.

[17] El-Hennawy A. M., Abd-Elwahab A. M., Abd-Elmaksoud A. M., El-Ozairy H. S., Boulis S. R. (2009). Addition of clonidine or dexmedetomidine to bupivacaine prolongs caudal analgesia in children. *British Journal of Anaesthesia*.

[18] Anand V. G., Thavamani A., Kannan M., Bridgit M. J. (2011). Effects of dexmedetomidine added to caudal ropivacaine in paediatric lower abdominal surgeries. *Indian Journal of Anaesthesia*.

[19] Xiang Q., Huang D. Y., Zhao Y. L. (2013). Caudal dexmedetomidine combined with bupivacaine inhibit the response to hernial sac traction in children undergoing inguinal hernia repair. *British Journal of Anaesthesia*.

[20] Lee C., Kim Y.-D., Kim J.-N. (2013). Antihyperalgesic effects of dexmedetomidine on high-dose remifentanil-induced hyperalgesia. *Korean Journal of Anesthesiology*.

[21] Jain G., Bansal P., Ahmad B., Singh D., Yadav G. (2012). Effect of the perioperative infusion of dexmedetomidine on chronic pain after breast surgery. *Indian Journal of Palliative Care*.

[22] Nama Sharanya B. S., Meenan D. R., Fritz W. T. (2010). The use of sub-anesthetic intravenous ketamine and adjuvant dexmedetomidine when treating acute pain from CRPS. *Pain Physician*.

[23] Li S.-S., Zhang W.-S., Yang J.-L., Xiong Y.-C., Zhang Y.-Q., Xu H. (2013). Involvement of protein Kinase B/Akt in analgesic effect of dexmedetomidine on neuropathic pain. *CNS Neuroscience and Therapeutics*.

[24] Farghaly H. S. M., Abd-Ellatief R. B., Moftah M. Z., Mostafa M. G., Khedr E. M., Kotb H. I. (2014). The effects of dexmedetomidine alone and in combination with tramadol or amitriptyline in a neuropathic pain model. *Pain Physician*.

[25] Lavand'homme P., De Kock M. (2006). The use of intraoperative epidural or spinal analgesia modulates postoperative hyperalgesia and reduces residual pain after major abdominal surgery. *Acta Anaesthesiologica Belgica*.

[26] Lauretti G. R., Rodrigues A. D. M., Gomes J. M. A., Dos Reis M. P. (2002). Epidural ketamine versus epidural clonidine as therapeutic for refractory neuropathic chronic pain. *Revista Brasileira de Anestesiologia*.

